# Pre-service teachers' insights on climate change and health in Kosovo: Exploring knowledge, attitudes, and practices

**DOI:** 10.1016/j.joclim.2025.100434

**Published:** 2025-03-20

**Authors:** Lira Ramadani, Susanne Lopez Lumbi, Zeqir Veselaj, Melanie Boeckmann

**Affiliations:** a Department of Global Health, Institute of Public Health and Nursing Research, University of Bremen, Germany (Grazer Straße 4, 28359 Bremen); b Faculty of Health Sciences, Bielefeld University, Germany (Universitätsstraße 25, 33615 Bielefeld); c Medical School OWL, AG Sustainable Environmental Health Sciences, Bielefeld University, Germany; d Faculty of Education, University of Prishtina University, Kosovo; e Department of Global Health, Institute of Public Health and Nursing Research, University of Bremen, Germany

**Keywords:** climate change, health literacy, pre-service teachers, knowledge, attitudes, practices, climate change education

## Abstract

**Introduction:**

As educators play a pivotal role in shaping the perspectives of future agents of change, it is crucial to assess their knowledge, attitudes, and practices regarding climate change, currently considered one of the biggest health emergencies.

**Methods:**

We conducted a cross-sectional survey with 137 students enrolled in teacher education programs at the Faculty of Education, University of Prishtina, Kosovo. Participants were approached via convenience sampling. Descriptive and inferential statistics were used for data analyses.

**Results:**

Our findings revealed misconceptions regarding the causes of climate change, with 64% incorrectly attributing climate change to natural processes or equal combined natural and human causes. Likewise, over 94% of the respondents were not aware of the scientific consensus on anthropogenic climate change. However, 97% reported that they think climate change currently is affecting the health of individuals at least a moderate amount. Most respondents identified illness from reduced air quality (95.6%) as an exacerbated health outcome due to climate change, while mental health conditions were perceived as the least important in connection to climate change (47.4%). A multiple linear regression model with age, gender, education level, mother's education, father's education, place of residence, attitudes and practices explained 44% of climate-health knowledge.

**Conclusion:**

The findings from this research could contribute to the development of targeted interventions and educational strategies aimed at enhancing pre-service teachers' knowledge of climate change and health-related challenges, thereby enabling them to effectively impart this knowledge to their future students.

## 1 Introduction

Climate change has profound implications on public health, impacting existing health threats and giving rise to new challenges [[1], [2], [3]]. The anticipated risks encompass various aspects of life, including extreme weather events, undernutrition, vector-borne diseases, waterborne diseases, and heat stress, these affect both vulnerable populations, including low-income populations, outdoor workers, women, children, the elderly, and individuals with chronic diseases or disabilities [4,5], and the broader community [6]. Considering the significant direct and indirect impacts of climate change on human health, increasing knowledge and essential skills are vital for effective mitigation and adaptation strategies. Framing climate change as a health issue has been proposed to emphasize its seriousness and urgency, altering the perception of this global phenomenon [7]. Additionally, recent research emphasizes shifting from “climate change literacy” to “climate and health literacy” to capture the health impacts [8], and therefore establishing specific educational goals becomes imperative. These include identifying local impacts, developing science-based curricula, and creating a network for communication and resource-sharing [9]. Enhancing climate health literacy, especially among school children, is crucial for fostering a generation capable of addressing the impacts of climate change [8,10].

Education, particularly through the lens of Education for Sustainable Development (ESD), has been identified as a crucial way to address the challenges posed by climate change [11,12], with the United Nations Educational, Scientific, and Cultural Organization (UNESCO) leading the effort for this integration into both formal and non-formal education [13]. Despite the acknowledged role of education in addressing climate change, various challenges persist. The successful integration of ESD relies heavily on teachers, who must demonstrate pro-environmental attitudes and behaviors while possessing sufficient knowledge for effective teaching [14]. Studies indicate inadequate and inaccurate education on climate change among students, with a considerable number of teachers facing difficulties in knowing how to teach these complex issues [15]. Barriers to teaching climate change are compounded by overcrowded curricula, a lack of interdisciplinary training, teacher reluctance to address its abstract nature [16], low levels of enthusiasm, and the use of traditional teaching methods [17,18]. In addition, studies have attributed the teachers’ inadequate knowledge and lack of confidence in teaching topics on climate change to insufficient background information in their university studies [16,19]. When teachers hold misconceptions, they are more inclined to transmit these inaccurate understandings to their students [15]. In this context, knowledge of the basic science of climate change among students enrolled in teacher education programs, or pre-service teachers (PSTs), is a factor that deserves attention, since it is a necessary requirement for developing pedagogically sound learning experiences for school students from the earliest years of school [20].

Increasing knowledge on climate change and health is vital in Kosovo, due to its susceptibility to impacts and low environmental knowledge among youth, as described below. The Western Balkans region, located in Southeast Europe, faces significant climate change impacts, including rising temperatures, altered precipitation patterns, and increased risks of droughts, floods, and forest fires [6,21]. Kosovo, particularly vulnerable due to resource mismanagement and inadequate policies, experiences exacerbated environmental issues, such as air pollution stemming largely from lignite coal burning and emissions from old cars [22,23]. One of the biggest concerns, air pollution, associated with over 3,000 premature deaths per 100,000 in 2020, is predicted to be exacerbated by climate change [24]. Recent research shows that climate change-related health outcomes in Kosovo, including heat-related illnesses, mental and physical harm from extreme weather, diseases, economic hardship, violence, dislocations, and service disruptions, are expected to worsen due to projected impacts. Children, with 23% living in poverty and lacking essential services, are particularly vulnerable to environmental hazards like extreme heat, storms, and pollution [25]. Despite these issues, studies reveal low environmental knowledge among youth and university students [[26], [27], [28]]. This is particularly concerning given that Kosovo has the youngest population in Europe, with 55% under 30 years old [29].

Given the global impact of climate change on public health, the education sector plays a key role in raising awareness and promoting sustainability for future generations. A World Health Organization survey revealed that out of 46 countries, almost half (46%), reported that inadequate research prevents national strategies related to health and climate change [30].

Until now, the evaluation of knowledge, attitudes, and practices concerning climate change and health among PSTs has been largely overlooked [31]. Prior studies focused predominantly on climate change in isolation [[20], [32], [33]], or targeted medical students learning health effects [[34], [35]], underscoring the importance of such evaluations given the imminent threat that climate change poses to public health and the role of PSTs as change agents [36].

## 2 Methodology

### 2.1 Survey design

**Fig. 1:**
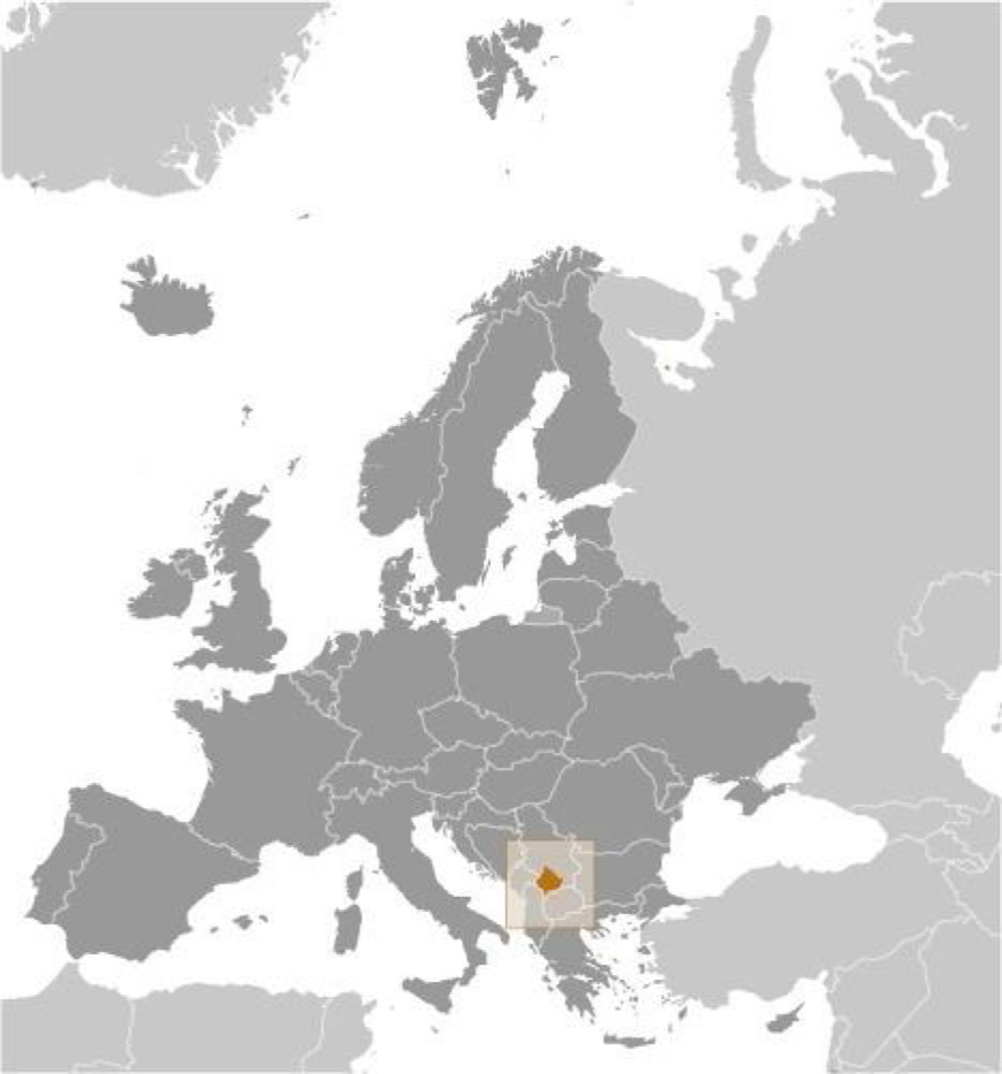
Location of Kosovo within Europe [39].

This research adopts a cross-sectional survey design to assess pre-service teachers' knowledge, perceptions, attitudes, and practices regarding climate change and health. The survey was conducted using LimeSurvey (Version 3.27.30+211,222) with students at the Faculty of Education - University of Prishtina - Kosovo, during March - April 2024. See Fig. 1 for the location of Kosovo within Europe.

PSTs are students in Bachelor's and Master's programs at the Faculty of Education, including Early Childhood Education, Primary Education, Pedagogy, and specialized programs in Educational Sciences and Subject Teaching (e.g., Math, Science, Literature). These programs prepare PSTs to teach various subjects and levels, from early childhood to secondary education [37]. PSTs may incorporate climate change into their teaching as part of the cross-curricular inclusion of Education for Sustainable Development (ESD) in curricula in Kosovo [38].

The survey includes multiple-choice and Likert scale questions to gather comprehensive data on general climate change knowledge (climate change causes, impacts, mitigation), climate change and health-related knowledge, attitudes and perceptions, as well as individual climate action or practices. Finally, demographic data was collected such as gender, age, place of residence, education level, and parents’ education level. Understanding the nuances of climate change, including perceptions, knowledge levels, and engagement, is influenced by demographic characteristics such as gender, age, and ethnicity [40].

The instrument was adapted from previous relevant surveys to fit the specific context of this study [35,[41], [42], [43], [44], [45], [46], [47], [48], [49], [50]]. The instrument was discussed with three experts (public health and education sciences experts with over 10 years of experience) in both English and Albanian. The final instrument contained 20 questions (available in the supplementary materials). In addition, a pilot test was conducted with 10 students, to confirm the clarity and internal consistency of the questions. The pilot test was successful (Cronbach's alpha= 0.869) as measured from the Likert scales in the attitude dimension; thus, no changes were made, and the responses were included in the final analysis. Based on the pilot testing, responders took around 20–25 minutes to complete the survey. The survey items, originally in English, were translated into Albanian and back-translated into English by two bilingual individuals to ensure accuracy.

### 2.2 Ethical Consideration

Informed consent was obtained from respondents, emphasizing voluntary participation, confidentiality, anonymity of data, length of survey, data use and protection and purpose of the study. The research was approved by the Bielefeld University Ethical Committee (No. 2024–031 of 2023/01/31).

### 2.3 Participants

Through convenience sampling, the researcher (LR) conducted in-person recruitment by visiting classrooms at the Faculty of Education, University of Prishtina. During these visits, a QR code linking to the online survey was shared with students. The open survey also was distributed via emails and social media. After excluding 63 incomplete surveys, we were left with 137 fully completed surveys, which were included in the analysis. To ensure survey quality, we provide all relevant information on the CHERRIES checklist (The Checklist for Reporting Results of Internet E-Surveys) [51] available as a supplementary file.

### 2.4 Data Analysis

Descriptive and inferential analyses were employed to quantify and analyze survey responses. Linear regression models were used to explore predictors of knowledge using R and R Studio. To this end, composite variables were created by select questions of the knowledge, attitude and practices dimensions. For the knowledge items (Question K8 - Supplementary Files), correct answers “Yes” were scored 1, while incorrect “No” or "Don't know" responses were scored 0, a common method in knowledge surveys [52]. A composite score (ranging from 0 to 13) was calculated based on 13 Likert scale items, reflecting the total number of correct responses, since all listed health outcomes are affected by climate change. Composite scores were also calculated for the attitude variable (Question A1- Supplementary Files), where each attitude statement was rated on a 5-point Likert scale with items marked as SA = 5, A = 4, N = 3, D = 2, and SD = 1. Similarly, for the practice variable (Question P1 - Supplementary Files), items reflecting favorable environmental actions were scored as 1 (if the practice was for environmental reasons) and 0 for no action or if the practice was done for other reasons. Assumptions for multiple linear regression were tested and met (Multiple Linear Regression and aGSIF values in the Supplementary Materials).

## 3 Results

Out of 137 students surveyed, most were aged between 18–20 years old (49.6%), and the majority were of Albanian ethnicity (98.5%). In addition, most of the participants were female (92.7%). There was a somewhat balanced number of participants in terms of place of residence, with 54% living in urban areas and 46% int rural areas. Urban areas are considered cities, towns and suburbs, whereas rural areas are villages [53]. Most participants were bachelor students (80.3%), whereas 19.7% were master's students.

Most (75%) of participants’ mothers had primary or secondary education as their highest level of education, while 16% have completed higher education (Professional, Bachelor’s, Master’s, or Doctoral degrees). Among fathers, 51% had primary or secondary education as their highest level of education, and 40.9% have attained higher education degrees.

### 3.1 General knowledge on climate change

In terms of the causes of climate change, 64.2% of the respondents answered incorrectly, stating that climate change is caused equally by both natural and human causes, or only from natural causes, whereas 35.8% answered correctly – that climate change is caused mainly by humans. When asked about the percentage of climate scientist that agree that climate change is human-caused, only 5.1 % of the respondents answered correctly (97–100%).

The energy sector was the most frequent choice as the greatest contributor to climate change at 78%, followed by waste landfills and incineration, and industry, both at 68%.

The most common climate change impact reported was glacier reduction (81.8%) while sea acidification received the fewest responses at 32.1%. Reduction of the use of vehicles (82.5%) was the most selected item as a mitigation measure, while improvement of technology garnered the fewest responses (27%) (Table 1).

**Table 1:** Climate change causes, impacts and mitigation knowledge.

**Over the last 150 years, climate change has been...**	**N=137**	**%**
Mainly caused by human factors*	49	35.8
Mainly a natural phenomenon	10	7.3
Caused equally by both human activities and natural changes in the environment	78	56.9
None of the above because climate change isn't happening	0	0
**To the best of your knowledge, what percentage of climate scientists think that human-caused climate change is happening?**	**N**	**%**
0–35 (%)	11	8.0
36–70 (%)	64	46.7
71–90 (%)	40	29.2
91 - 96 (%)	15	10.9
97 - 100 (%)*	7	5.1
**What contributes most to climate change? (Choose all that apply)**	**N**	**%**
Transportation	74	54
Buildings and Construction Sector	40	29.2
Ozone Hole	75	54.7
Industry	94	68.6
Agriculture	26	19
Waste landfills and waste incineration	94	68.6
Energy Sector (electricity and heating production)	107	78.1
None of them	1	0.7
**Select the impacts of climate change. (Choose all that apply)**	**N**	**%**
Crop decline	59	43.1
Local droughts/floods	94	68.6
Sea level rise	62	45.3
Biodiversity loss	73	53.3
Increase in extreme weather events (heatwave, cold spell, floods)	98	71.5
Water shortage	68	49.6
Glacier reduction	112	81.8
Sea acidification	44	32.1
Health risks	76	55.5
None of them	0	0
**Select the mitigation methods of climate change. (Choose all that apply)**	**N**	**%**
Reduce using vehicles	113	82.5
Reduce/sort garbage	98	71.5
Save electricity	99	72.3
Plant trees	100	73.0
Save water	71	51.8
Improve technology	37	27.0
None of them	1	0.7

*correct answers

### 3.2 Climate change and health-related knowledge and attitudes

Most respondents, 97%, reported that they think climate change is currently affecting the health of individuals at a moderate amount or a great deal. In terms of the self-reported knowledge on climate change and health impacts, only 5% reported they are “very knowledgeable”, whereas the majority reported they are “moderately knowledgeable" regarding climate change health impacts (57.7%) (Table 2).

**Table 2:** Self-reported knowledge and perceptions on climate change and health.

**How knowledgeable do you feel about the association between climate change and health impacts?**	N=137	%
Not at all knowledgeable	6	4.4
Modestly knowledgeable	45	32.8
Moderately knowledgeable	79	57.7
Very knowledgeable	7	5.1
**How much, if at all, do you think climate change is currently affecting the health of individuals?**	**N**	**%**
Not at all	0	0.0
Only a little	4	2.9
A moderate amount	88	64.2
A great deal	45	32.8

When asked specifically about the specific health outcomes exacerbated by climate change, most respondents (95.6%) reported that illness due to reduced outdoor air quality would be exacerbated, followed by increased poverty due to economic hardship (91.2 %).

The least exacerbated health outcomes were reported as anxiety, depression, or other mental health conditions (47.4%); and violence, conflict, and/or resulting dislocation (48.9 %).

**Fig. 2:**
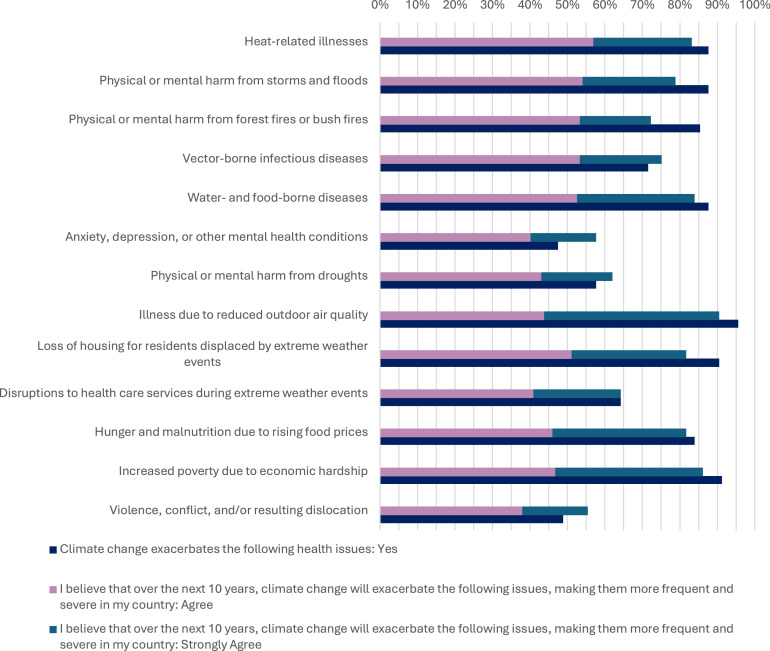
Climate change and health knowledge and attitudes of participants.

In terms of the attitudes on the exacerbation of health-related issues by climate change over the next 10 years in Kosovo, the same responses were given. Illness due to air pollution was again the most reported (43.8% Agree; 46.7 Strongly Agree), followed by increased poverty due to economic hardship (46.7% Agree; 39.4% Strongly Agree). Mental health conditions were reported as the least likely to be exacerbated (40.1% Agree; 17.5% Strongly Agree), followed by violence/conflict/relocation (38% Agree; 17.5% Strongly Agree) (Fig. 2).

**Fig. 3:**
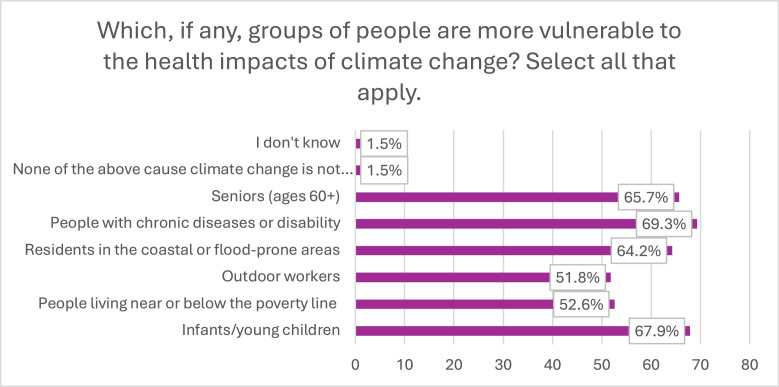
Reported vulnerable groups.

The most vulnerable group identified by the respondents were people with chronic diseases and disability at 69.3%, whereas the least vulnerable were outdoor workers at 51.8%. Only 1.5% of the respondents responded that there are no vulnerable groups as climate change is not happening (Fig. 3).

### 3.3 Pre-service teachers’ practices - individual climate actions

When asked about pro-environmental practices, 80% of respondents reported that they do not follow a vegan/vegetarian diet, followed by 54.7 % reporting taking no action in sorting waste/recycling. In contrast 62.8% reported that they pay attention to water consumption, for environmental reasons and 59.1% reported that they “turn off lights/appliances when not in use” for environmental reasons (Table 3).

**Table 3:** Individual climate actions of pre-service teachers.

Statement	Mostly for environmental reasons		Mostly for other reasons		No action	
	**n**	***%***	***n***	**%**	**n**	**%**
I walk/use a bicycle	16	11.7	71	51.8	50	36.5
I use public transport	20	14.6	108	78.8	9	6.6
I sort waste/recycle	50	36.5	12	8.8	75	54.7
I use energy-efficient lightbulbs	47	34.3	34	24.8	56	40.9
I turn off lights/appliances not in use	81	59.1	55	40.1	1	0.7
I pay attention to energy efficiency and electricity consumption	60	43.8	56	40.9	21	15.3
I pay attention to water consumption	86	62.8	44	32.1	7	5.1
When possible, I buy local produce	58	42.3	50	36.5	29	21.2
I grow fruits/vegetables for my own needs	53	38.7	53	38.7	31	22.6
I don't consume meat (I follow a vegan/vegetarian diet)	6	4.4	21	15.3	110	80.3

### 3.4 Multiple linear regression model for individual climate action

We conducted a multiple linear regression analysis with age, gender, own education level, father's education, mother's education level, place of residence, attitudes and practices as predictors. The model explains 44% of the variability of the knowledge variable (adjusted R^2^ = 0.44, F (19, 116) = 6.49, p < 0.001). Notably, respondents who preferred not to disclose their mother's education level exhibited a significant decrease in knowledge scores compared to those with a bachelor's degree (β = -4.85; p = 0.02). Similar significant decreases were observed for respondents whose mothers had primary education (β = -2.21; p = 0.01) or secondary education (β = -2.25; p = 0.005) compared to those with a bachelor's degree. Conversely, respondents whose fathers had secondary education showed a significant increase in knowledge scores compared to those with a bachelor's degree (β = 1.43; p = 0.009). Overall, attitudes and practices also emerged as significant predictors, positively influencing knowledge scores (β = 0.19; p < 0.001 and β = 0.25; p = 0.0001, respectively).

## 4 Discussion

Our study provides insights into the knowledge, attitudes and practices of PSTs at the University of Prishtina, Kosovo, on climate change and health. While almost all (97%) respondents think climate change is currently affecting the health of individuals a moderate amount or a great deal, they generally believe climate change has and will have a lower impact on mental health and societal issues, despite poverty, compared to physical health-related issues (heat-related illnesses, air quality illness and water- and food-borne diseases).

The multiple linear regression model with factors age, gender, education level, mother's education, father's education, place of residence, attitudes and practices explained 44% of the climate-health knowledge. Findings suggest that parental education levels, particularly maternal, shape respondents' knowledge, *with an unexpected higher score for those whose fathers had secondary over bachelor’s education*, while positive attitudes and practices also correlate with higher knowledge.

In addition, the noted misconceptions about climate change highlight the need for targeted educational initiatives to improve climate and health literacy among PSTs.

### 4.1 Knowledge and Individual Climate Action

We observed a significant positive association between participants' knowledge about climate change and their adoption of environmentally-friendly practices. This aligns with the results of other studies showing that greater knowledge and awareness of climate change across various groups [[54], [55], [56]] positively influences the adoption of sustainable practices and pro-environmental behaviors.

However, Liu et al. [57] argue that environmental knowledge does not influence behavior directly, yet is a crucial distal factor that mediates pro-environmental behavior via environmental attitudes and behavioral intentions. In contrast to the findings of this study, other research reports that high environmental awareness/attitudes do not always correspond with pro-environmental behaviors, often referred to as an attitude-behavior gap [[58], [59], [60]]. Reasons for this are not fully explored, but it is suggested that many environmentally unfriendly behavior are often habits, which are hard to change [61]. Another explanation is the low-cost hypothesis, stating that stronger environmental attitudes weaken when behaviors become costly [62].

In terms of individual climate action, most participants (80.3%) reported that they eat meat (take no action in following a vegan/vegetarian diet) followed by no action taken in sorting waste or recycling (54.8%). A possible explanation could also be that meat is a staple of the diet in Kosovo and the Balkans [63]. It must also be noted that our large sample of females may have influenced the statistics, as they are likely to consume less meat than men [[64], [65]]. Although young generations have proven to be key drivers of global climate action and awareness-raising initiatives, exemplified by the Fridays for Future movement [[66], [67], [68]], their climate change awareness and behavior are often contradictory, with a global perspective but inconsistent personal actions [69]. Energy efficiency actions, like turning off appliances or paying attention to electricity consumption and efficiency, are often done "mostly for other reasons" (40.1%; 40.9%), likely due to energy poverty in Kosovo [70].

### 4.2 Climate Science Misconceptions

Although the respondents reported a high level of self-reported knowledge regarding climate change and health, the results revealed some misconceptions regarding climate change causes among participants. For instance, 64.2% of the participants incorrectly attribute climate change to natural processes or equal natural and human causation, as opposed to attributing climate change mainly to anthropogenic causes (35.8%). Similarly, over 94% of the respondents were not aware of the scientific consensus on human-caused climate change. Climate scientists largely agree that human activity is the primary driver of climate change. A comprehensive study estimates this consensus at 97% [71], while more recent findings suggest it could be as high as 99% [72]. Our results are consistent with Boon (2010) [73], who also found that self-reported familiarity with topics like climate change did not correlate with individuals' scientific knowledge.

The survey responses highlight discrepancies compared to the IPCC's assessment of climate change contributors. For example, the IPCC (2022) [74] identifies the energy sector as the largest contributor (34%), followed by industry (24%), AFOLU (Agriculture, Forestry, and Other Land Use) (22%), transport (15%), and lastly, buildings (6%). The survey also ranks the Energy Sector the highest, with respondents ranking Industry and Waste landfills/incineration equally as the second-largest contributors, while Agriculture ranked lowest. However, the survey's classification of the sectors includes different boundaries that do not fully align with IPCC's categories. It is important to note that the relative size of each sector depends on how boundaries are defined [74,75]. Additionally, 54%, of respondents incorrectly attributed climate change to the ozone hole, a misconception consistent with previous research on PSTs [32,[74], [75], [76], [77], [78]] and students from other majors [64,[65], [79]], who often confuse the scientific basis of climate phenomena like ozone depletion and climate change. This underscores the critical need for enhancing PSTs’ scientific knowledge and correcting these misconceptions through comprehensive and accurate climate change education, including specially designed learning materials that confront students with their incorrect ideas [[80], [81], [82]].

### 4.3 Gender and Learning Needs

The demographic analysis from the University of Prishtina for the 2023/24 academic year highlights that the teaching profession remains predominantly female, with 424 (88.33%) female and 56 (11.67%) male students [83]. These statistics were expected as the teaching profession is known to be dominated by women [84]. This demographic trend is significant as our study found that the mother's education level predicts knowledge of climate change and health, consistent with findings that link it to climate knowledge and eco-friendly practices [85,86]. Given the role of parental education, particularly mothers, this underscores opportunities for intergenerational knowledge transfer. Tackling climate change education among PSTs is especially important as women generally exhibit higher levels of environmental concern and are more likely to engage in pro-environmental behaviors [87]. Educational programs should harness this predisposition by creating curricula that address climate change and health topics and empower women to lead in climate change advocacy and education.

### 4.4 Limitations

The survey was conducted at one single institution using convenience sampling, which may restrict the applicability of the findings to wider groups of students in Kosovo and beyond. There is a possibility of selection bias as respondents were self-selected, and some may have had a strong inclination to share opinions about climate change and health. Another limitation of this study is the demographic imbalance in the sample, including the high proportion of female participants (92.7%). This may limit the generalizability of the findings, as responses might differ in a more balanced sample. Future research could aim for larger sample sizes that would facilitate identification of differences that may exist among these groups.

## 5 Conclusion

While PSTs recognize the impact of climate change on physical health, they underestimate its effects on mental health and societal issues and hold misconceptions about climate science. These gaps underscore the need for targeted educational initiatives to enhance climate-health literacy. Effective curricula should clearly differentiate between issues like ozone depletion and climate change, address broad health impacts, and promote responsible behaviors. Preparing and empowering PSTs as knowledge multipliers and agents of change is essential for equipping future generations with the knowledge and skills to tackle climate change and its impacts effectively.

## CRediT authorship contribution statement

**Lira Ramadani:** Writing – review & editing, Writing – original draft, Visualization, Methodology, Investigation, Formal analysis, Conceptualization. **Susanne Lopez Lumbi:** Writing – review & editing, Formal analysis. **Zeqir Veselaj:** Writing – review & editing. **Melanie Boeckmann:** Writing – review & editing, Supervision.

## Declaration of competing interest

The authors declare that they have no known competing financial interests or personal relationships that could have appeared to influence the work reported in this paper.

## Appendix Supplementary materials

Image, application 1

Image, application 2

## References

[1] Ebi KL, Balbus JM, Luber G, Bole A, Crimmins A, Glass G, et al. Human Health. In: Reidmiller DR, Avery CW, Easterling DR, Kunkel KE, Lewis KLM, Maycock TK, et al., editors. Impacts, risks, and adaptation in the united states: fourth national climate assessment, Volume II. Washington, DC, USA: U.S. Global Change Research Program; 2018, p. 539–571. doi: 10.7930/NCA4.2018.CH14.

[2] Luber G, Knowlton K, Balbus J, Frumkin H, Hayden M, Hess J, Melillo JM, Richmond TC, Yohe GW (2014). Climate change impacts in the united states: the third national climate assessment.

[3] Watts N, Amann M, Arnell N, Ayeb-Karlsson S, Belesova K, Boykoff M (2019). The 2019 report of The Lancet Countdown on health and climate change: ensuring that the health of a child born today is not defined by a changing climate. The Lancet.

[4] Levy BS, Patz JA. (2015). Climate change, Human rights, and Social justice. Ann Glob Health.

[5] Balbus JM, Malina C. (2009). Identifying vulnerable subpopulations for climate change health effects in the United States. J Occup Environ Med.

[6] IPCC. Summary for policymakers. Cambridge: Intergovernmental Panel on Climate Change; 2021.

[7] Adlong W, Dietsch E. (2015). Environmental education and the health professions: framing climate change as a health issue. Environ Educ Res.

[8] Limaye V, Grabow M, Stull V, Patz J. (2020). Developing A definition of climate and health literacy. Health Aff (Millwood).

[9] Shaman J, Knowlton K. (2018). The need for climate and health education. Am J Public Health.

[10] Grabow ML, Stull VJ, Hahn MB, Limaye VS. (2023). A blueprint for strengthening climate and health literacy through professional adaptability. Front Public Health.

[11] Agbedahin AV. Sustainable development, Education for Sustainable Development, and the 2030 Agenda for Sustainable Development: emergence, efficacy, eminence, and future. Sustainable Development. 2019;27(4):669–80. doi: 10.1002/sd.1931.

[12] Mochizuki Y, Bryan A. (2015). Climate change education in the context of education for Sustainable Development: rationale and principles. J Educ Sustain Dev.

[13] UNESCO (2013).

[14] Esa N. (2010). Environmental knowledge, attitude and practices of student teachers. Int Res Geogr Environ Educ.

[15] Hung CC. Climate change education: knowing, doing and being. Routledge; 2022.

[16] Fortner RW. (2001). Climate change in school: where does it fit and how ready are we?. Can J Environ Educ CJEE.

[17] Bozdoğan AE. (2009). An investigation on Turkish prospective primary school teachers’ perceptions about global warming. World Appl Sci J.

[18] Ocal A, Kisoglu M, Alas A, Gurbuz H. (2011). Turkish prospective teachers’ understanding and misunderstanding on global warming. Int Res Geogr Environ Educ.

[19] Berger P, Gerum N, Moon M. (2015). Roll up your sleeves and get at it!” Climate change education in teacher education. Can J Environ Educ.

[20] Boon HJ. (2020). Pre-service teachers and climate change: A stalemate?. Aust J Teach Educ Online.

[21] Vuković A, Vujadinović Mandić M. Study on climate change in the Western Balkans region. Sarajevo, Bosnia and Herzegovina: Regional Cooperation Council Secretariat; 2018. ISBN: 978-9926-402-09-9. Accessed 27 January 2024. Available from: https://www.rcc.int/pubs/62/study-on-climate-change-in-the-western-balkans-region

[22] KEPA (2022).

[23] Shala K, Dorri A (2021). Analysis of the motor vehicle fleet as a way to reduce air pollution in the Republic of Kosovo. Int J Innov Technol Interdiscip Sci.

[24] European Environment Agency (2022).

[25] UNICEF (2024). https://www.unicef.org/kosovoprogramme/media/5501/file/CLAC_ENG.pdf.pdf.

[26] Lindemann-Matthies P, Hyseni M. (2009). Perception and knowledge of environmental issues, in particular biodiversity by stakeholders and laypersons in Kosovo- A case study. J Int Environ Appl Sci.

[27] Veselaj Z, Torkar G. (2017). The acceptability of teachers’ Value related statements about sustainable development and climate change among Non-science and science major students from Kosovo. Eur J Sustain Dev.

[28] Ymeri P, Bytyqi P, Shala-Abazi A, Fetoshi O, Millaku F, Fogarassy C. (2023). University students’ environmental world views based on the new environmental paradigm (NEP) scale: a case study from Kosovo. Environ Dev Sustain.

[29] Ministry of Culture, Youth and sports. State Strategy For Youth 2024-2032. Republic of Kosovo: Republic of Kosovo Government; n.d.

[30] WHO (2021).

[31] Akiba M. (2011). Identifying program characteristics for preparing pre-service teachers for diversity. Teach Coll Rec.

[32] Nyarko SC, Petcovic HL. (2021). Ghanaian preservice science teachers’ knowledge of ozone depletion and climate change, and sources of their knowledge. Int J Sci Educ.

[33] Lambert JL, Bleicher RE. (2013). Climate change in the preservice teacher's mind. J Sci Teach Educ.

[34] Rybol L, Nieder J, Amelung D, Hachad H, Sauerborn R, Depoux A, et al. Integrating climate change and health topics into the medical curriculum – a quantitative needs assessment of medical students at Heidelberg University in Germany 2023. 10.3205/ZMA001618. PMC10291352 37377571

[35] Yang L, Liao W, Liu C, Zhang N, Zhong S, Huang C. (2018). Associations between knowledge of the causes and perceived impacts of climate change: a cross-sectional survey of medical, public health and nursing students in universities in China. Int J Environ Res Public Health.

[36] Winter V, Kranz J, Möller A. (2022). Climate change education challenges from two different perspectives of change agents: perceptions of school students and pre-service teachers. Sustainability.

[37] Universiteti i Prishtinës Fakulteti i Edukimit. Programet, https://edukimi.uni-pr.edu/page.aspx?id=1,3; 2024 [accessed 13 December 2024].

[38] Veselaj Z, Krasniqi Z. (2014). Mapping of education for sustainable development in the new curriculum of Kosovo and challenges of implementation. SGEM Conference Proceedings.

[39] CIA (2025). https://www.cia.gov/the-world-factbook/countries/kosovo/locator-map/.

[40] Wolf J, Moser SC. (2011). Individual understandings, perceptions, and engagement with climate change: insights from in-depth studies across the world. WIREs Clim Change.

[41] Breakey S, Starodub R, Nicholas PK, Wong J. (2023). A cross-sectional study to assess faculty and student knowledge of climate change and health: readiness for curricular integration. J Adv Nurs.

[42] Kotcher J, Maibach E, Miller J, Campbell E, Alqodmani L, Maiero M (2021). Views of health professionals on climate change and health: a multinational survey study. Lancet Planet Health.

[43] Salem MR, Hegazy N, Thabet Mohammed AA, Mahrous Hassan E, Saad Abdou MM, Zein MM (2022). Climate change-related knowledge and attitudes among a sample of the general population in Egypt. Front Public Health.

[44] Sarfaty M, Mitchell M, Bloodhart B, Maibach E. (2014). A survey of African American physicians on the health effects of climate change. Int J Environ Res Public Health.

[45] UNICEF (2021).

[46] Wachholz S, Artz N, Chene D. (2014). Warming to the idea: university students’ knowledge and attitudes about climate change. Int J Sustain High Educ.

[47] Wang Y, Zhang X, Li Y, Liu Y, Sun B, Wang Y (2022). Knowledge, attitude, risk perception, and health-related adaptive behavior of primary school children towards climate change: A cross-sectional study in China. Int J Environ Res Public Health.

[48] Leiserowitz A, Smith N, Marlon J (2011).

[49] Leviston, Z. & Walker, I.A. Baseline survey of Australian attitudes to climate change: PRELIMINARY REPORT. 2011.

[50] Nam AH, Lee S., Reimers FM (2021). Educ. clim. change, cham.

[51] Eysenbach G. (2004). Improving the quality of web surveys: the checklist for reporting results of internet E-surveys (CHERRIES). J Med Internet Res.

[52] Connor M, Siegrist M. (2010). Factors influencing people's acceptance of gene technology: the role of knowledge. Health Expectations, Naturalness, and Social Trust. Sci Commun.

[53] Mela A. Urban Areas. In: Michalos AC, editor. Encycl. qual. life Well- Res., Dordrecht: Springer Netherlands; 2014, p. 6826–8. 10.1007/978-94-007-0753-5_3122.

[54] Li W, Ruiz-Menjivar J, Zhang L, Zhang J. (2021). Climate change perceptions and the adoption of low-carbon agricultural technologies: evidence from rice production systems in the Yangtze River Basin. Sci Total Environ.

[55] Zhang X, Khachatryan H, Knuth M. (2021). Relating knowledge and perception of sustainable landscape practices to the adoption intention of environmentally friendly landscapes. Sustainability.

[56] Ma L, Shahbaz P, ul Haq S, Boz I (2023). Exploring the moderating role of environmental education in promoting a clean environment. Sustainability.

[57] Liu P, Teng M, Han C. (2020). How does environmental knowledge translate into pro-environmental behaviors?: the mediating role of environmental attitudes and behavioral intentions. Sci Total Environ.

[58] ElHaffar G, Durif F, Dubé L. (2020). Towards closing the attitude-intention-behavior gap in green consumption: A narrative review of the literature and an overview of future research directions. J Clean Prod.

[59] Kollmuss A, Agyeman J. (2002). Mind the gap: why do people act environmentally and what are the barriers to pro-environmental behavior?. Environ Educ Res.

[60] Park HJ, Lin LM. (2020). Exploring attitude–behavior gap in sustainable consumption: comparison of recycled and upcycled fashion products. J Bus Res.

[61] Linder N, Giusti M, Samuelsson K, Barthel S. (2022). Pro-environmental habits: an underexplored research agenda in sustainability science. Ambio.

[62] Diekmann A, Preisendörfer P. (2003). Green and Greenback: the behavioral effects of environmental attitudes in low-cost and high-cost situations. Ration Soc.

[63] Jasar D, Curcic B., Gostin A-I, Bogueva D, Kakurinov V (2021). Nutritional health aspects of food in the balkans.

[64] Rosenfeld DL, Tomiyama AJ. (2021). Gender differences in meat consumption and openness to vegetarianism. Appetite.

[65] Rousset S, Deiss V, Juillard E, Schlich P (2005). Droit-Volet S. Emotions generated by meat and other food products in women. Br J Nutr.

[66] Huttunen J, Albrecht E. (2021). The framing of environmental citizenship and youth participation in the Fridays for Future Movement in Finland. Fenn - Int J Geogr.

[67] Marquardt J. (2020). Fridays for Future's disruptive Potential: an inconvenient youth between moderate and radical ideas. Front Commun.

[68] Parth A-M, Weiss J, Firat R, Eberhardt M. (2020). How dare you!”—The influence of fridays for future on the political attitudes of young adults. Front Polit Sci.

[69] Skeirytė A, Krikštolaitis R, Liobikienė G. (2022). The differences of climate change perception, responsibility and climate-friendly behavior among generations and the main determinants of youth's climate-friendly actions in the EU. J Environ Manage.

[70] Deutsche Gesellschaft für Internationale Zusammenarbeit (GIZ) GmbH (2022). https://www.giz.de/en/downloads/giz2023-en-factsheet-energy-poverty.pdf.

[71] Cook J, Oreskes N, Doran PT, Anderegg WRL, Verheggen B, Maibach EW (2016). Consensus on consensus: a synthesis of consensus estimates on human-caused global warming. Environ Res Lett.

[72] Lynas M, Houlton BZ, Perry S. (2021). Greater than 99% consensus on human caused climate change in the peer-reviewed scientific literature. Environ Res Lett.

[73] Boon H. (2010). Climate change? Who knows? A comparison of secondary students and pre-service teachers. Aust J Teach Educ.

[74] Dhakal S, Minx JC, Toth FL, Abdel-Aziz A, Figueroa Meza MJ, Hubacek K, Shukla PR, Skea J, Slade R, Al Khourdajie A, van Diemen R, McCollum D (2022). Climate change 2022: mitigation of climate change. contribution of working group iii to the sixth assessment report of the intergovernmental panel on climate change.

[75] Lamb WF, Grubb M, Diluiso F, Minx JC. (2022). Countries with sustained greenhouse gas emissions reductions: an analysis of trends and progress by sector. Clim Policy.

[76] Khalid T. (2003). Pre-service high school teachers’ Perceptions of three environmental phenomena. Environ Educ Res.

[77] Khalid T. (2001). Pre-service teachers’ misconceptions regarding three environmental issues. Can J Environ Educ CJEE.

[78] Papadimitriou V. (2004). Prospective primary teachers’ understanding of climate change, greenhouse effect, and ozone layer depletion. J Sci Educ Technol.

[79] Milovanovic J, Shealy T, Godwin A (2022). Senior engineering students in the USA carry misconceptions about climate change: implications for engineering education. J Clean Prod.

[80] Competente RJT. (2019). Pre-service teachers’ inclusion of climate change education. Int J Eval Res Educ IJERE.

[81] Walz KA, Kerr SC. (2007). Holes” in student understanding: addressing prevalent misconceptions regarding atmospheric environmental chemistry. J Chem Educ.

[82] Sellmann D, Bogner FX., Leal Filho W (2012). Clim. change sustain. use water resour..

[83] University of Prishtina (2024). https://app.powerbi.com/view?r=eyJrIjoiMWJlOGU1MzgtMTBiNC00ZWM1LThkYWEtZmU1ZTRjYjNlNzBlIiwidCI6ImMwMzRjM2I0LWQwMmItNDM2MS04M2YwLTNiMWI5ZWE0MzcwYyIsImMiOjh9&pageName=ReportSection.

[84] Drudy S. (2008). Gender balance/gender bias: the teaching profession and the impact of feminisation. Gend Educ.

[85] Akpan EP, Falaye FV. (2009). Parental educational background and supportiveness in students’ preference for technical and vocational education. Niger J Appl Psychol.

[86] Falaye FV, Okwilagwe EA. (2016). Assessing the senior school students’ Knowledge, attitude and practices related to climate change: implications for curriculum review and teacher preparation. J Int Soc Teach Educ.

[87] May AM, McGarvey MG, Gustafson CR, Mieno T. (2021). Gender, environmental issues and policy: an examination of the views of male and female economists. Ecol Econ.

