# Supplementary material for: Pre-service teachers' insights on climate change and health in Kosovo: Exploring knowledge, attitudes, and practices
Source: J Clim Chang Health. 2025 Mar 20;22:100434. doi: 10.1016/j.joclim.2025.100434 (PMC12851167; doi:10.1016/j.joclim.2025.100434)
Supplement: Supplementary file 1 [file mmc1.docx]

| **Original question(s)** | **Reference** | **Question number** | **Adapted question**  (Correct answers, where applicable, marked in green) |
| --- | --- | --- | --- |
| **Climate change knowledge and perceptions (Climate change causes, impacts and mitigation knowledge)** | | | |
| Do you think climate change over the past 150 years is...   - Caused entirely by human activities - Caused mostly by human activities - Caused about equally by human activities and natural changes in the environment - Caused mostly by natural changes in the environment. - Caused entirely by natural changes in the environment - None of the above because climate change isn’t happening | Sarfaty et al., 2014 [1] | **K1** | Over the last 150 years, climate change has been...   - Mainly caused by human factors - Mainly a natural phenomenon. - Caused equally by both human activities and natural changes in the environment - None of the above because climate change isn’t happening |
| Assuming global warming is happening, do you think it is…   - Caused mostly by human activities - Caused by both human and natural changes (vol.) - Caused mostly by natural changes in the environment - None of the above because global warming isn’t happening - Other - Don’t know (vol.) | Leiserowitz et al., 2011 [2] |  |  |
| To the best of your knowledge, what percentage of climate scientists think that human-caused climate change is happening?   - 0-35 (%) - 36-70 (%) - 71-90 (%) - 91 - 96 (%) - 97 - 100 (%)   *Slider bar question* | Kotcher et al., 2021 [3] | **K2** | To the best of your knowledge, what percentage of climate scientists think that human-caused climate change is happening?   - 0-35 (%) - 36-70 (%) - 71-90 (%) - 91 - 96 (%) - 97 - 100 (%) |
| What do you think contributes the most to climate change? (multiple answers possible) The industry   - The energy sector (electricity production) - Transport and traffic - Construction - Heating of households with non-environmental energy sources - Industry heating (heating of small and larger companies) - Incineration of waste at illegal landfills - Agriculture (e.g., burning of stubble and agricultural crops) - Other (please write it down) - I don’t know/Prefer not to say | UNICEF, 2021 [4] | **K3** | What contributes most to climate change? (Choose all that apply)   - Industry - Energy sector (electricity and heating production)* - Transportation - Building and Construction sector - Ozone hole - Waste landfills and waste incineration - Agriculture - None of them   *biggest contributor according to IPCC (2022)[5] |
| How much does each of the following contribute to global warming?   - Cars and trucks - Burning fossil fuels for heat and electricity - Toxic wastes - Deforestation - The hole in the ozone layer - Nuclear power plants - Aerosol spray cans - The sun - Acid rain - Volcanic eruptions - Cows - The space program   *Response options: A lot; Some; A little; Not at all; don’t know* | Leiserowitz et al., 2011 [2] |  |  |
| Select the impacts of climate change. (Choose all that apply)   - Crop decline - Water shortage - Local droughts/floods - Glacier reduction - Sea level rise - Sea acidification - Biodiversity loss - Health risks - Increase in extreme weather events (heatwave, cold spell, floods) - None of them | Wang et al., 2022 [6] | **K4** | Select the impacts of climate change. (Choose all that apply)   - Crop decline - Local droughts/floods - Sea level rise - Biodiversity loss - Increase in extreme weather events (heatwave, cold spell, floods) - Water shortage - Glacier reduction - Sea acidification - Health risks - None of them |
| Select the mitigation methods of climate change. (Choose all that apply)   - Reduce using vehicles - Plant trees - Reduce/sort garbage - Save water - Save electricity - Improve technology - None of them | Wang et al., 2022 [6] | **K5** | Select the mitigation methods of climate change. (Choose all that apply)   - Reduce using vehicles - Reduce/sort garbage - Save electricity - Plant trees - Save water - Improve technology - None of them |
| **Climate change and health knowledge and perceptions** | | | |
| How knowledgeable do you feel about the association between climate change and health impacts?   - Very knowledgeable - Moderately knowledgeable - Modestly knowledgeable - Not at all knowledgeable | Sarfaty et al., 2014 [1] | **K6** | How knowledgeable do you feel about the association between climate change and health impacts?   - Very knowledgeable - Moderately knowledgeable - Modestly knowledgeable - Not at all knowledgeable |
| How knowledgeable do you feel about the association between climate change and health impacts?   - Not at all knowledgeable - Modestly knowledgeable - Moderately knowledgeable - Very knowledgeable | Breakey et al.,2023 [7] |  |  |
| How much, if at all, do you think climate change is currently affecting the health of individuals?   - Not at all - Only a little - A moderate amount - A great deal | Breakey et al. 2023 [7] | **K7** | How much, if at all, do you think climate change is currently affecting the health of individuals?   - Not at all - Only a little - A moderate amount - A great deal |
| Do you agree each following issue as a health impact of climate change?   - Vector-borne infectious disease - Flooding-related displacement - Mental health conditions - Air quality-related illness - Food-borne disease - Disruption of health care services during extreme weather events - Water-borne infectious disease - Heat-related illness - Cold-related illness - Water-availability illness - Malnutrition - Other climatic change health-related impacts   *Response Options: Yes; No; Don’t Know* | Yang et al., 2018 [8] | **K8** | Climate change exacerbates the following health issues:   - Heat-related illness - Physical or mental harm from storms or floods - Physical or mental harm from forest fires or bush fires - Vector-borne infectious diseases - Water- and food-borne diseases - Anxiety, depression, or other mental health conditions - Physical or mental harm from droughts - Illness due to reduced air quality - Loss of housing for residents displaced by extreme weather events - Disruption of health care services during extreme weather events - Hunger and malnutrition due to rising food prices - Increased poverty due to economic hardship - Violence, conflict, and/or resulting dislocation   *Response Options: Yes*; No; Don’t Know*  *Correct for all response options |
| How much, if at all, has climate change already adversely affected these health issues in your country?   - Heat-related illnesses - Physical or mental harm from storms and floods - Physical or mental harm from forest fires or brush fires - Vector-borne infectious diseases - Water- and food-borne diseases - Anxiety, depression, or other mental health conditions - Physical or mental harm from droughts - Illness due to reduced outdoor air quality - Loss of housing for residents displaced by extreme weather events - Disruptions to health care services during extreme weather events - Hunger and malnutrition due to rising food prices - Increased poverty due to economic hardship - Violence, conflict, and/or resulting dislocation   *Response Options: Not at all; Only a little; A moderate mount; A great deal; Don’t know* | Kotcher et al., 2021 [3] |  |  |
| Which, if any, of the following groups will disproportionately experience any negative health effects from climate change? (check all that apply).   - Young children ages 0 to 4 - Older children ages 5 to 17 - Young adults ages 18 to 39 - Middle-aged adults ages 40 to 60 - Older adults ages 60+ - People with chronic diseases - People living near or below the poverty line - People of color - None of the above because climate change isn’t happening | Sarfaty et al., 2014 [1] | **K9** | Which, if any, groups of people are more vulnerable to the health impacts of climate change? Select all that apply.   - Infant/young children - People living near or below the poverty line - Outdoor workers - Residents in coastal or flood-prone areas - People with chronic diseases or disability - Seniors (ages 60+) - None of the above because climate change isn’t happening - I don’t know |
| In your opinion, who is more vulnerable to the effects of global warming/ climate change? (you can choose more than one answer)   - Infant/young children - The poor and the disadvantaged - Outdoor workers - People living with light or sensitive skin - Residents on the coast or in flood-prone areas - The sick, disabled, obese, and with low immunity - Everyone - No group is more vulnerable than others | Salem et al.,2022 [9] |  |  |
| **Climate change and health attitudes** | | | |
| Over the next 10 years, do you think climate change will make these health issues in your country more frequent or severe, less frequent or severe, or do you think they will remain largely unchanged?   - Heat-related illnesses - Physical or mental harm from storms and floods - Physical or mental harm from forest fires or brush fires - Vector-borne infectious diseases - Water- and food-borne diseases - Anxiety, depression, or other mental health conditions - Physical or mental harm from droughts - Illness due to reduced outdoor air quality - Loss of housing for residents displaced by extreme weather events - Disruptions to health care services during extreme weather events - Hunger and malnutrition due to rising food prices - Increased poverty due to economic hardship - Violence, conflict, and/or resulting dislocation   *Response options: More frequent or severe; Less frequent or severe; Will remain unchanged; Don’t know* | Kotcher et al., 2021 [3] | **A1** | I believe that over the next 10 years, climate change will exacerbate the following issues, making them more frequent and severe in my country:   - Heat-related illness - Physical or mental harm from storms or floods - Physical or mental harm from forest fires or bush fires - Vector-borne infectious diseases - Water- and food-borne diseases - Anxiety, depression, or other mental health conditions - Physical or mental harm from droughts - Illness due to reduced air quality - Loss of housing for residents displaced by extreme weather events - Disruption of health care services during extreme weather events - Hunger and malnutrition due to rising food prices - Increased poverty due to economic hardship - Violence, conflict, and/or resulting dislocation   *Response Options: Strongly Agree; Agree; Neutral; Disagree, Strongly Disagree* |
| **Individual practices (individual climate action)** | | | |
| How often do you practice the following activities?   - I walk - I ride a bicycle/scooter to work - I use public transport - I sort waste - I use energy-efficient lightbulbs - I consume organic food - I collect garbage from public areas - I pay attention to water consumption - I grow fruits/vegetables for my own needs - I don’t use plastic bags - I avoid using plastic - I donate clothes - I compost - I pay attention to energy efficiency and electricity consumption - I eat meat - I talk about ecology with my family and friends   *Response options: Regularly (almost every day); Often (a few times a week); Sometimes (two to three times a month); Rarely (once a*  *month, a few times a year); I don’t practice it at all* | UNICEF, 2021 [4] | **P1** | Below is a set of activities for which you must determine whether you practice them or not, and what is the motive for practicing them.  Activity:   - I walk/use the bike - I use public transport - I sort waste/recycle - I use energy-efficient light bulbs - Turn off lights/devices when not in use - I pay attention to energy efficiency - I pay attention to water consumption - When possible, buy local produce - I grow vegetables/fruits for my needs - I don't consume meat (I follow a vegan/vegetarian diet)   *Response options: No action; Mostly for environmental reasons; Mostly for other reasons* |
| - I have a vegetarian (or vegan) diet - I have taken part in a political campaign about an environmental issue - I have contacted a government member about climate change - I am on Green Power electricity - I live within 5 kilometers of my workplace - I usually walk/cycle/carpool/take public transport to work - I have a front-loading washing machine - I try to buy products that are second-hand - I grow a lot of my own vegetables - Most of my cleaning products are environmentally friendly - I have switched to products that are more environmentally friendly - Where possible, I buy products that are made locally - I have reduced the amount of gas and/or electricity I use around the house - I will usually try to fix things rather than replace them - I have reduced the amount of water I use around the house and garden - I recycle my household waste - I switch lights off around the house whenever possible   *Response options: No action; Mostly for environmental reasons; Mostly for other reasons* | Leviston, Z. & Walker, I.A., 2011 [10] |  |  |

| **Demographic Data** | D1. What is your age? ____________________ Years (please type in numbers)  D2. What is your gender?   - - - Male     - Female     - Non-binary/Diverse     - Prefer not to say   D3. What is your place of Residence?   - - - Urban     - Rural   D4. What is your ethnicity?   - - - Albanian     - Serbian     - Turkish     - Roma     - Ashkali     - Egyptian     - Bosniak     - Gorani     - Other     - Prefer not to answer   D5a. What is your mother’s highest level of education?   - No formal education - Primary education - Secondary education - Vocational or technical education - Bachelor's degree - Master's degree - Doctoral degree - Prefer not to answer   D5b. What is your father’s highest level of education?   - No formal education - Primary education - Secondary education - Vocational or technical education - Bachelor's degree - Master's degree - Doctoral degree - Prefer not to answer   D6. In which program are you currently enrolled?   - Bachelor’s - Master’s |
| --- | --- |

References:

[1] Sarfaty M, Mitchell M, Bloodhart B, Maibach E. A Survey of African American Physicians on the Health Effects of Climate Change. IJERPH 2014;11:12473–85. https://doi.org/10.3390/ijerph111212473.

[2] Leiserowitz A, Smith N, Marlon J. American Teens’ Knowledge of Climate Change. New Haven, CT: Yale University; 2011.

[3] Kotcher J, Maibach E, Miller J, Campbell E, Alqodmani L, Maiero M, et al. Views of health professionals on climate change and health: a multinational survey study. The Lancet Planetary Health 2021;5:e316–23.

[4] UNICEF. Knowledge, attitudes and practices study on climate change and the environment (Education). North Macedonia: UNICEF; 2021.

[5] IPCC. Emissions Trends and Drivers. Climate Change 2022 - Mitigation of Climate Change. 1st ed., Cambridge University Press; 2023, p. 215–94. https://doi.org/10.1017/9781009157926.004.

[6] Wang Y, Zhang X, Li Y, Liu Y, Sun B, Wang Y, et al. Knowledge, Attitude, Risk Perception, and Health-Related Adaptive Behavior of Primary School Children towards Climate Change: A Cross-Sectional Study in China. International Journal of Environmental Research and Public Health 2022;19:15648.

[7] Breakey S, Starodub R, Nicholas PK, Wong J. A cross‐sectional study to assess faculty and student knowledge of climate change and health: Readiness for curricular integration. Journal of Advanced Nursing 2023;79:4716–31. https://doi.org/10.1111/jan.15729.

[8] Yang L, Liao W, Liu C, Zhang N, Zhong S, Huang C. Associations between knowledge of the causes and perceived impacts of climate change: a cross-sectional survey of medical, public health and nursing students in universities in China. International Journal of Environmental Research and Public Health 2018;15:2650.

[9] Salem MR, Hegazy N, Thabet Mohammed AA, Mahrous Hassan E, Saad Abdou MM, Zein MM. Climate change-related knowledge and attitudes among a sample of the general population in Egypt. Front Public Health 2022;10:1047301. https://doi.org/10.3389/fpubh.2022.1047301.

[10] Leviston, Z. & Walker, I.A. Baseline Survey of Australian attitudes to climate change: PRELIMINARY REPORT. 2011.

Table 1. Participants’ demographic data

| Characteristics | N=137 | % |
| --- | --- | --- |
| Age (years) | | |
| Under 18 | 1 | 0.7 |
| 18-20 | 68 | 49.6 |
| 21-25 | 45 | 32.8 |
| Above 25 | 17 | 12.4 |
| Not given | 6 | 4.4 |
| Ethnicity | | |
| Albanian | 135 | 98.5 |
| Turkish | 1 | 0.7 |
| RAE | 1 | 0.7 |
| Gender | | |
| Female | 127 | 92.7 |
| Male  Diverse | 8  0 | 5.8  0 |
| Prefer not to say | 2 | 1.5 |
| Place of residence | | |
| Urban | 74 | 54.0 |
| Rural | 63 | 46.0 |
| Mother’s highest level of education | | |
| No formal education | 1 | 0.7 |
| Primary education | 39 | 28.5 |
| Secondary education | 64 | 46.7 |
| Professional education | 10 | 7.3 |
| Bachelor diploma | 8 | 5.8 |
| Master diploma | 2 | 1.5 |
| Doctoral degree | 2 | 1.5 |
| Prefer not to say | 11 | 8 |
| Father’s highest level of education | | |
| No formal education | 1 | 0.7 |
| Primary education | 9 | 6.6 |
| Secondary education | 61 | 44.5 |
| Professional education | 24 | 17.5 |
| Bachelor diploma | 22 | 16.1 |
| Master diploma | 6 | 4.4 |
| Doctoral degree | 4 | 2.9 |
| Prefer not to say | 10 | 7.3 |
| Own current level of education | | |
| Bachelor | 110 | 80.3 |
| Master | 27 | 19.7 |

| Variables | Adjusted R^2^ | F* | β | Std. error | p-value | 95% confidence intervals for β | |
| --- | --- | --- | --- | --- | --- | --- | --- |
|  |  |  |  |  |  | 2.5% | 97.5% |
|  | 0.44 | 6.49 |  |  | **<0.001** |  |  |
| Intercept |  |  | -0.52 | 1.85 | 0.78 | -4.18 | 3.14 |
| age |  |  | 0.05 | 0.05 | 0.35 | -0.06 | 0.16 |
| gender_male^1^ |  |  | 0.57 | 0.90 | 0.53 | -1.21 | 2.34 |
| gender_prefer not to say^1^ |  |  | 0.19 | 1.48 | 0.90 | -2.73 | 3.12 |
| residence_urban^2^ |  |  | 0.26 | 0.36 | 0.47 | -0.45 | 0.96 |
| mother_Prefer not to answer^3^ |  |  | -4.85 | 2.09 | **0.02** | -8.98 | -0.72 |
| mother_Primary education^3^ |  |  | -2.21 | 0.83 | **0.01** | -3.86 | -0.57 |
| mother_Secondary education^3^ |  |  | -2.25 | 0.78 | **0.005** | -3.80 | -0.69 |
| mother_Vocational or technical education^3^ |  |  | -1.12 | 0.95 | 0.24 | -3.00 | 0.76 |
| mother_Master’s degree^3^ |  |  | -1.77 | 1.59 | 0.27 | -4.92 | 1.38 |
| mother_Doctoral degree^3^ |  |  | -1.09 | 2.15 | 0.61 | -5.35 | 3.17 |
| father_Prefer not to answer^4^ |  |  | 2.46 | 2.09 | 0.24 | -1.68 | 6.61 |
| father_Primary education^4^ |  |  | 1.18 | 0.84 | 0.16 | -0.48 | 2.83 |
| father_Secondary education^4^ |  |  | 1.43 | 0.54 | **0.009** | 0.36 | 2.49 |
| father_Vocational or technical education^4^ |  |  | 0.30 | 0.60 | 0.62 | -0.89 | 1.49 |
| father_Master’s degree^4^ |  |  | -1.02 | 0.95 | 0.28 | -2.90 | 0.85 |
| father_Doctoral degree^4^ |  |  | 0.04 | 1.44 | 0.98 | -2.81 | 2.90 |
| Education_Master^5^ |  |  | -0.50 | 0.68 | 0.46 | -1.84 | 0.84 |
| attitudes |  |  | 0.19 | 0.03 | **<0.001** | 0.14 | 0.24 |
| practices |  |  | 0.25 | 0.07 | **0.001** | 0.10 | 0.39 |

Table 2. Results of the multiple linear regression model

* F on 19 and 116 degrees of freedom; ^1^reference group: gender_female; ^2^reference group: residence_rural; ^3^reference group: mother_bachelor degree; ^4^reference group: father_bachelor degree; ^5^reference group: education_bachelor

Table 3. aGSIF values for each variable

| **Variables** | **aGSIF*** |
| --- | --- |
| age | 1.50 |
| gender | 1.16 |
| residence | 1.10 |
| mother | 1.40 |
| father | 1.37 |
| education | 1.65 |
| attitudes | 1.12 |
| practices | 1.21 |

*aGSIF = adjusted generalized standard error inflation factor
