# Supplementary material for: Pre-service teachers' insights on climate change and health in Kosovo: Exploring knowledge, attitudes, and practices
Source: J Clim Chang Health. 2025 Mar 20;22:100434. doi: 10.1016/j.joclim.2025.100434 (PMC12851167; doi:10.1016/j.joclim.2025.100434)
Supplement: Supplementary file 2 [file mmc2.docx]

| **Item Category** | **Checklist Item** | **Explanation** |
| --- | --- | --- |
| **Design** | Describe survey design | Cross-sectional survey: The study assessed pre-service teachers' knowledge, perceptions, attitudes, and practices regarding climate change and health. It was conducted using LimeSurvey (Version 3.27.30+211222) at the Faculty of Education, University of Prishtina, during March-April 2024. |
| **IRB (Institutional Review Board) approval and informed consent process** | IRB approval | Approved by Bielefeld University Ethical Committee: Approval No. 2024-031 of 2023/01/31. |
|  | Informed consent | Emphasized voluntary participation, confidentiality, anonymity of data, survey length, data use and protection, and study purpose. |
|  | Data protection | Data were protected, ensuring confidentiality and anonymity. Questionnaires were anonymous. LimeSurvey has their server in the European Union which ensures data protection under EU-GDPR. |
| **Development and pretesting** | Development and testing | Survey instrument designed based on a review of existing surveys on climate change and health. The survey was validated by three experts, reviewed, and pre-tested with the research team and their networks. A pilot test with 10 students confirmed clarity and internal consistency (Cronbach’s alpha = 0.869). |
| **Recruitment process and description of the sample having access to the questionnaire** | Open survey versus closed survey | Open survey: Distributed via email, social media and QR code distribution via direct in-person classroom contact. This approach allowed a broader participation but makes it impossible to calculate an exact response rate. |
|  | Contact mode | Distributed via email, social media and direct contact. |
|  | Advertising the survey | Advertised through emails, social media platforms and direct contact. |
| **Survey administration** | Web/E-mail | Administered via LimeSurvey. A link/QR code was distributed via e-mail, social media and direct contact to potential participants. |
|  | Context | Conducted online among pre-service teachers at the University of Prishtina. |
|  | Mandatory/voluntary | Voluntary participation. |
|  | Incentives | No incentives provided. |
|  | Time/Date | March and April 2024. |
|  | Randomization of items or questionnaires | Question order was not randomized. |
|  | Adaptive questioning | Yes: Some questions were used as filter questions, answered only by certain participants. |
|  | Number of Items | The survey has a total of 28 questions, but 19 were used for this study (see survey questions in the supplementary files) |
|  | Number of screens (pages) | 6 pages |
|  | Completeness check | Survey instrument was pre-tested, and pilot tested for clarity and consistency. |
|  | Review step | Backward navigation was allowed. |
| **Response rates** | Unique site visitor | Not applicable in the context of a survey distributed via email and social media. |
|  | View rate (Ratio of unique survey visitors/unique site visitors) | Not applicable for email/social media distributed surveys. |
|  | Participation rate (Ratio of unique visitors who agreed to participate/unique first survey page visitors) | Not applicable; not calculable due to the nature of distribution. |
|  | Completion rate (Ratio of users who finished the survey/users who agreed to participate) | 137 completed surveys out of 200 total responses (137 completed + 63 incomplete), implying a completion rate of 68.5%. |
| **Preventing multiple entries from the same individual** | Cookies used | Cookies were not used. |
|  | IP check | Since the participants are university students who typically use the campus network, their IP addresses may be duplicated. Therefore, we chose not to track participants' IP addresses. |
|  | Log file analysis | N/A |
|  | Registration | N/A |
| **Analysis** | Handling of incomplete questionnaires | 63 incomplete surveys were excluded from the analysis |
|  | Questionnaires submitted with an atypical timestamp | The system's cache mechanism, with "participants may save and resume later" enabled, allowed participants to continue the survey after exiting accidentally. As a result, we did not exclude subjects based on completion time. However, only data submitted before the set deadline was included in the analysis. |
|  | Statistical correction | Descriptive and inferential statistical methods were used, including a regression model to explore predictors of knowledge. A composite variable was created for knowledge, attitudes, and practices.  Missing data (variable age) was filled using data imputation. |
